# Selective Pressures on Human Cancer Genes along the Evolution of Mammals

**DOI:** 10.3390/genes9120582

**Published:** 2018-11-28

**Authors:** Alberto Vicens, David Posada

**Affiliations:** 1 Department of Biochemistry, Genetics and Immunology, University of Vigo, 36310 Vigo, Spain; avicens@uvigo.es; 2 Biomedical Research Center (CINBIO), University of Vigo, 36310 Vigo, Spain; 3 Galicia Sur Health Research Institute, 36310 Vigo, Spain

**Keywords:** positive selection, somatic evolution, germline evolution, dN/dS

## Abstract

Cancer is a disease driven by both somatic mutations that increase survival and proliferation of cell lineages and the evolution of genes associated with cancer risk in populations. Several genes associated with cancer in humans, hereafter cancer genes, show evidence of germline positive selection among species. Taking advantage of a large collection of mammalian genomes, we systematically looked for signatures of germline positive selection in 430 cancer genes available in COSMIC. We identified 40 cancer genes with a robust signal of positive selection in mammals. We found evidence for fewer selective constraints—higher number of non-synonymous substitutions per non-synonymous site to the number of synonymous substitutions per synonymous site (dN/dS)—and higher incidence of positive selection—more positively selected sites—in cancer genes bearing germline and recessive mutations that predispose to cancer. This finding suggests a potential association between relaxed selection, positive selection, and risk of hereditary cancer. On the other hand, we did not find significant differences in terms of tissue or gene type. Human cancer genes under germline positive selection in mammals are significantly enriched in the processes of DNA repair, with high presence of Fanconi anaemia/Breast Cancer A (FA/BRCA) pathway components and T cell proliferation genes. We also show that the inferred positively selected sites in the two genes with the strongest signal of positive selection, i.e., *BRCA2* and *PTPRC*, are in regions of functional relevance, which could be relevant to cancer susceptibility.

## 1. Introduction

Cancer is a genomic disease caused by mutations in genes that control normal cell functions, in particular growth and division. A fundamental goal of cancer genomics is identifying mutations that confer a selective advantage to the cell and increase survival and proliferation, the so-called driver mutations, as well as the genes carrying the driver mutations in each tumor, known as driver genes or cancer genes. Although traditionally the focus has been put on somatic driver mutations, those that appear during an individual lifetime as cells divide and grow, there are also germline mutations in the human population that predispose to cancer [1]. Today, more than 500 human cancer genes have been identified, of which approximately 90% contain somatic mutations and 20% bear germline mutations [2,3,4]

Several studies have identified a number of human cancer genes undergoing germline positive selection among species. Clark et al. revealed a strong evidence of positive selection on oncogenes and tumor suppressor genes in the chimpanzee lineage [5]. Nielsen et al. identified an elevated number of tumor suppressor and apoptosis genes under strong positive selection in humans and chimpanzees [6]. Subsequent genome-wide screenings in mammals unveiled positive selection in genes with roles in immunity and reproduction, but also related with apoptosis and cancer [7,8]. Given the recurrent observation of positive selection acting on human cancer genes across species, several authors have proposed that evolutionary pressures affecting organismal fitness, such as sexual selection, pathogen–host interactions, parent–offspring conflict, or maternal–fetal conflict, could lead to increased cancer risk in humans as a pleiotropic effect [6,7,8,9,10]. Another intriguing matter is whether the germline evolution of human cancer genes is influenced by their different characteristics, like genetic type, main tissue, or inheritance mode. For example, in hominoids it was observed that tumor suppressor genes (TSGs) tend to accumulate more non-synonymous substitutions than oncogenes, suggesting that the former are subjected to more relaxed purifying selection due to their recessive effects [11].

To address these questions in more detail, we carried out a comprehensive analysis of the evolution of 430 human cancer genes in mammals, after applying stringent criteria for evolutionary analysis. Using the ratio of number of non-synonymous substitutions per non-synonymous site to the number of synonymous substitutions per synonymous site (dN/dS), we identified 40 human cancer genes under putative positive selection in mammals. These genes are functionally enriched in DNA repair and immunity and are associated with hereditary cancer and recessive effects.

## 2. Materials and Methods

### 2.1. Cancer Genes

We retrieved 574 single-copy genes associated with human cancer from the Cancer Gene Census (CGC) project [4] of the COSMIC repository (https://cancer.sanger.ac.uk/cosmic, accession date: 5 March 2018). We only collected genes classified into Tier 1, which refers to genes with a documented activity relevant to cancer. The catalogue of the retrieved cancer genes, along with information about their function and associated mutations, is shown in Table S1.

### 2.2. Sequence Data Collection

We downloaded a representative human DNA sequence for each cancer gene from the Ensembl Genes database (Release 91, human genome version GRCh38.p12) using BioMart (accession date: 8 March 2018). For each human gene, we chose a single isoform on the basis of the following criteria, in the indicated order: GENCODE validation, APRIS annotation as principal 1, best transcript support level (TSL), and longer transcript. We discarded 22 genes whose best TSL was less than 1 (Table S2). Using the selected human isoform as a reference, we downloaded the corresponding orthologues from 32 mammalian genomes (Table S3, Figure S1) using the Bioconductor package BiomaRt [12]. When more than one ortholog was obtained for a given species, we chose the isoform with the best orthology confidence score. We discarded 17 genes for which less than 15 mammal orthologues were found (Table S2), so the final number of retrieved ortholog groups was 535 (Table S4).

### 2.3. Multiple Sequence Alignment

We aligned the coding sequences for each ortholog group using MACSE [13], a program that accounts for frameshifts and stop codons. The resulting multiple sequence alignments were further refined with TrimAl [14], removing taxa and sites with more than 60% gaps across rows and columns, respectively. After trimming, we discarded 71 genes that contained less than 10 orthologues (Table S2), in order to maximize the statistical power for the selection analyses, ending up with a list of 464 genes.

### 2.4. Estimation of Phylogenetic Trees

We inferred maximum likelihood (ML) gene trees for the 464 genes using Randomized Acelerated Maximum Likelihood-Next generation (RAxML-NG). All reconstructions were performed using the general time reversible substitution model [15] with gamma-distributed rate variation among sites [16]. For each gene, we obtained 10 starting trees using randomized stepwise addition parsimony. We assessed nodal support using 100 bootstrap replicates [17]. To minimize the impact of estimation errors and incomplete lineage sorting in subsequent analyses, we discarded 27 genes whose estimated tree topologies were quite distinct (normalized Robinson–Foulds (RF) distance ≥ 0.6) from a species tree assembled for 19 mammals with a well-known phylogenetic position (Figure S1). We calculated the RF distances with ETE3-compare [18].

### 2.5. Codon-Based Selection Models

We estimated nonsynonymous (dN) and synonymous (dS) substitution rates using the program codeml of the PAML package v4.9c [19] for 437 mammal gene trees. Because dS saturation decreases the power of detecting positive selection in codon-based models [20], we further discarded seven genes with an estimated dS > 15 (Table S2). To estimate the global dN/dS ratios for each of the remaining 430 genes, we used the one-ratio (M0) model, which assumes the same dN/dS for all branches in the gene tree and across sites. To identify genes under putative positive selection, we carried out three different tests. First, we compared different site-models using two likelihood ratio tests (LRTs): M1a (neutral) versus M2a (selection), and M8 (beta selection) versus M8a (beta neutral) [21,22]. The resulting *p*-values were adjusted for multiple testing using the Benjamini–Hochberg procedure [23] with a family-wise significance level of 0.05. In addition, we also tested for evidence of episodic positive selection using BUSTED [24], as implemented in Hyphy [25]. In order to be very stringent, only genes inferred to be under positive selection by the three tests were finally considered to be positively selected genes (PSGs). For those genes in which the LRT was significant, we considered as positively selected sites (PSS) those with a Bayes Empirical Bayes (BEB) posterior probability > 0.95 of having a dN/dS > 1 under both M2a and M8 [26].

### 2.6. Gene Ontology Enrichment Analysis

To identify enriched Gene Ontology (GO) terms in the PSGs, we used GOrilla [27]. We compared the list of 40 PSGs with a background list of the 574 cancer genes from CGC. We searched for significant GO terms (*p*-value < 0.01) in the three available ontologies: biological process, cellular component, and molecular function.

### 2.7. Pathogenic Germline Mutations

We retrieved the list of pathogenic germline variants for Breast cancer type 2 susceptibility (*BRCA2)* protein from the study published by the TCGA PanCanAtlas Germline Working Group [28].

### 2.8. Comparison of dN/dS Ratios across COSMIC Categories

We compared the M0 dN/dS ratios obtained across four different CGC–COSMIC classifications: mutation type, inheritance, tissue type, and cancer role. To test for significant dN/dS and proportion of PSGs differences between and among categories, we performed ANOVAs (for multiple comparisons) and *t*-tests (for pairwise comparisons) using the ggpubr package for R [29]. We adjusted the *p*-value for multiple pairwise comparisons using the Benjamin–Hochberg procedure. To compare the proportion of genes under putative positive selection across groups, we applied the chi-squared test (*p* < 0.05) function (chisq.test) implemented in R.

## 3. Results

After multiple processing steps and stringent criteria (see Materials and Methods), we finally assessed the selective pressures along the mammal phylogeny on 430 human cancer genes. Multiple sequence alignments included 11–32 taxa and were 108–4984 nt long (Table S5).

### 3.1. Long-Term Selective Pressures on Human Cancer Genes

**Figure 1:**
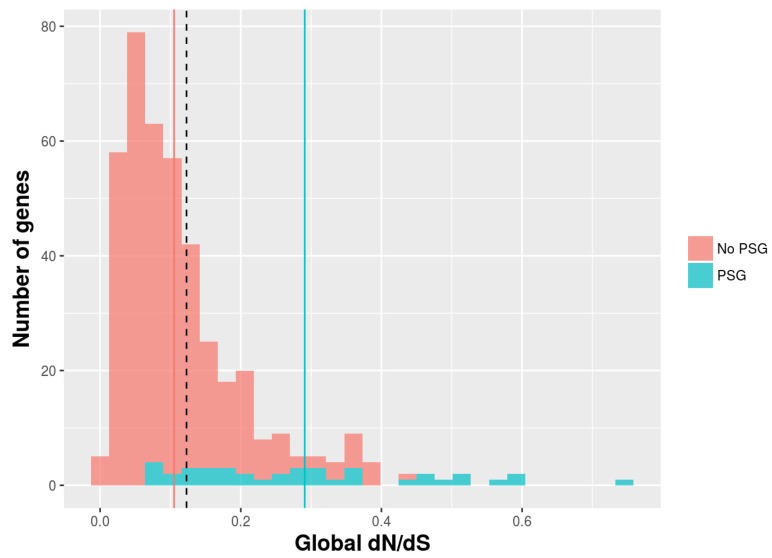
Distribution of global number of non-synonymous substitutions per non-synonymous site to the number of synonymous substitutions per synonymous site (dN/dS) estimates. Dashed, red, and blue vertical lines indicate mean dN/dS for all (0.122), positively selected (0.290), and not positively selected genes (0.105), respectively.

The mean dN/dS for the 430 cancer genes examined was 0.122. The LRTs among site-specific dN/dS models were significant for 56 (M2a) and 61 (M8) genes, while BUSTED was significant for 357 genes, in all three tests after correcting for multiple testing (*p*-adj < 0.05) (Table S5). Within these, 40 genes were identified with M2, M8, and BUSTED, and therefore considered to be PSGs (Table 1; Figure 1). All these genes showed at least one PSS under M2a or M8 (BEB > 0.95) (Table S5).

### 3.2. Comparison of Selection Estimates across Functional Categories

**Figure 2:**
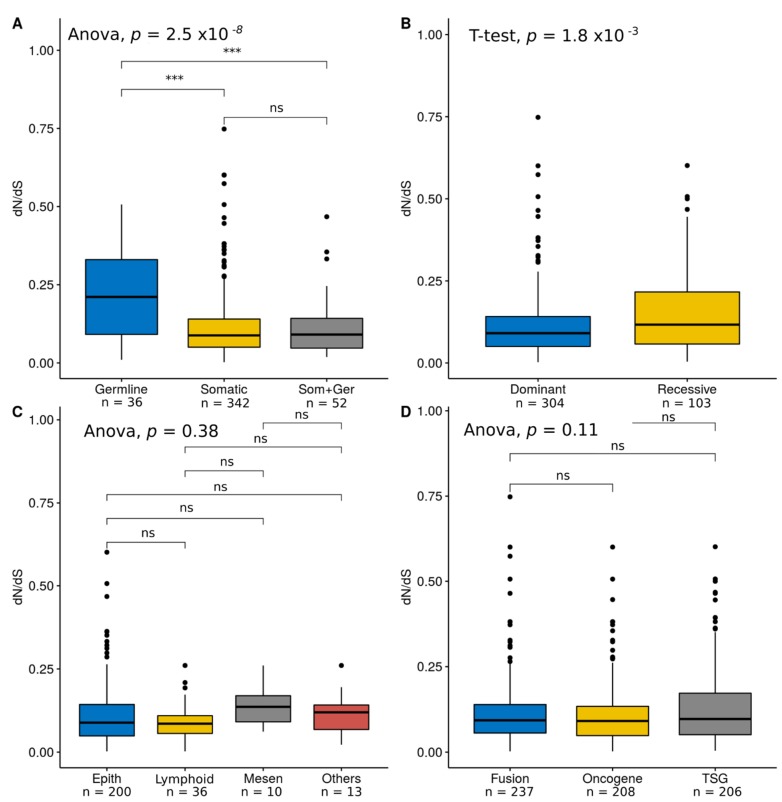
Global dN/dS across COSMIC categories. (**A**) Mutation type; (**B**) inheritance; (**C**) tissue type, and (**D**) cancer role; *p*-values are shown for each comparison: (ns): *p*-value > 0.01; (**): *p*-value < 0.01; (***): *p*-value < 0.001.

We compared global dN/dS values across COSMIC categories. Genes bearing only germline mutations (i.e., associated with hereditary cancer) showed significantly higher dN/dS estimates than genes with only somatic mutations (i.e., associated with sporadic cancer) or with both somatic and germline mutations (Figure 2A), mainly due to a significantly increase in dN (Figure S2). We also observed higher dN/dS values for cancer genes associated with recessive mutations than for cancer genes with dominant mutations (Figure 2B), again due to a significantly increase in dN (Figure S2). We noticed that these two mutational categories are not independent, as 33 out of 34 (97%) genes with germline mutations are associated with recessive inheritance. On the other hand, the global dN/dS estimates were not significantly different among tissue types (epithelial, lymphoid, mesenchymal, and others) (Figure 2C) or cancer role (fusion genes, oncogenes, and TSGs) (Figure 2D).

**Figure 3:**
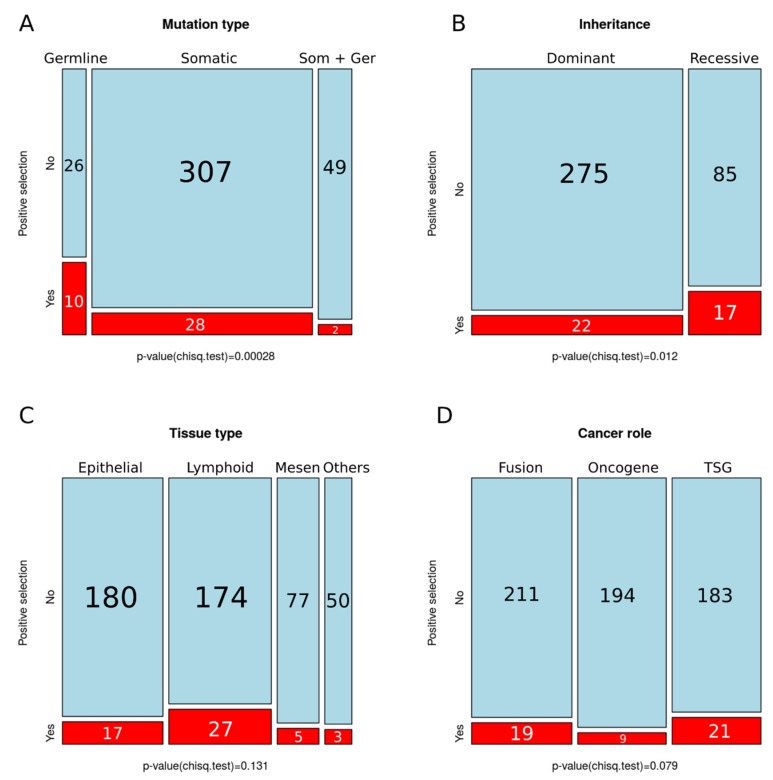
Number of positively selected genes across COSMIC categories. (**A**) Mutation type; (**B**) inheritance; (**C**) tissue type, and (**D**) cancer role; *p*-values for chi-squared tests are shown underneath (significance *p* < 0.05).

We also compared the proportion of PSGs across COSMIC categories, observing a significant increase in the germline (Figure 3A) and recessive categories (Figure 3B). We did not detect significant differences in the proportion of PSGs among tissue types (Figure 3C) or cancer role (Figure 3D).

Because a longer gene is more likely to have more spurious PSS than a shorter gene, we assessed whether the significant patterns just described were influenced by differences in protein length among categories. For this, we performed three statistical analyses. First, we compared protein length with global dN/dS, without detecting a significant correlation (Figure S3A). Second, we compared the protein length of PSGs and non-PSGs, again without observing significant differences (Figure S3B). Third, we compared the protein length among mutational categories (Figure S3C). Here, genes carrying germline mutations were not different from genes carrying only somatic mutations, but genes bearing both mutations were significantly larger; anyway, this did not interfere with our selection analyses. In addition, we contrasted the number of PSSs, normalized by sequence length, across COSMIC categories for both M2a and M8 models. We did not find significant differences in the proportion of PSSs for any functional category, regardless of the model (Figure S4).

### 3.3. Functional Enrichment of Positively Selected Cancer Genes

The 40 PSGs were enriched in biological processes associated with DNA repair (*p*-value = 2.63 × 10^−4^) and the regulation of T cell proliferation (*p*-value = 4.1 × 10^−4^). We did not identify significant enrichment of GO terms for molecular function or cellular component. Among DNA repair genes, we detected a high presence of members of the Fanconi Anemia Complementation Group (*FANCC*, *FANCD2*, *FANCG*, *FANCA*, and *FANCE*), which participate in homologous recombination DNA repair [30], and genes involved in double-strand break repair (*BRCA2*, *BRIP1*, *BARD*, *BLM*, *CHEK2*, and *PALB2*). In addition to the enrichment of genes involved in regulation of T cell proliferation, we observed a high proportion of PSGs related to immunity (Table 1).

### 3.4. Functional Relevance of Positively Selected Sites in Cancer Genes

**Figure 4:**
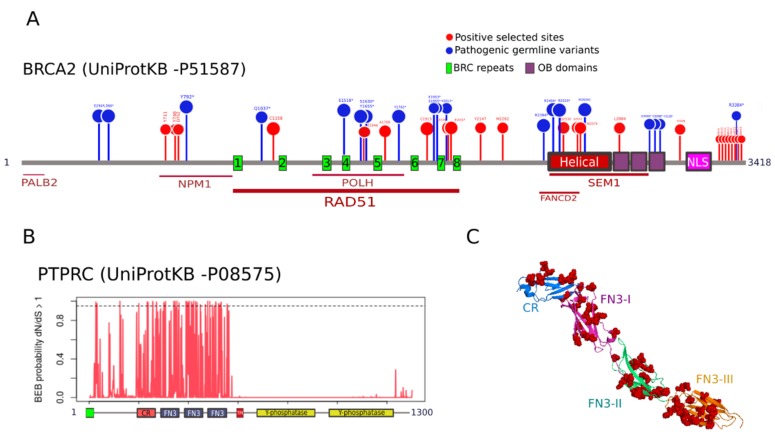
Positive selected sites in BRCA2 and PTPRC. (**A**) Mapping of positively selected sites (PSSs; red) and cancer predisposition variants (missensense and stop-gained; blue) in human *BRCA2*. Proteins interacting with different domains of BRCA2 are indicated underneath. Domain abbreviations: H: Helical domain; OB: Oligonucleotide Binding; NLS: Nuclear Localization Sequence; (**B**) Site-specific posterior probabilities of dN/dS > 1 in PTPRC, under the selection model M2a, are represented on the y-axis. The domain scheme is represented in the x-axis. The dashed line indicates the significance level for positive selected sites (Bayes Empirical Bayes (BEB) prob = 0.95). Domain abbreviations: CR: cysteine-rich; FN3 type 3: fibronectin 3; TM: transmembrane; (**C**) Crystal structure of the extracellular region of PTPRC (PDB ID: 5fmv, Chang et al., 2016), showing CR and FN3 domains with different colors. PSSs are shown as red spheres.

To understand the functional relevance of PSSs, we mapped their location in the two genes with the highest number of PSS, namely, *BRCA2* and *PTPRC*. *BRCA2* is a gene involved in double-strand break repair whose deficiency leads to hereditary breast and ovarian cancer [30]. We identified 16 and 25 PSSs under M2a and M8, respectively (Table S5). These two sets were nested, so we mapped the 25 M8 residues on human BRCA2 protein scheme (Figure 4A). We found that three selected residues (positions 711, 748, and 800) are located in the binding region of nucleophosmin (NPM) [31]. Six PSSs (1158, 1646, 1708, 1913, 2035, and 2037) are distributed along the BRC repeats that bind to RAD51 [32]. Among these, residues 1646 and 1708 sit in the interaction region with the polymerase Eta (POLH) [33]. We noticed that, in position 1913, 11 out of 27 species, including humans, have a Cys residue, whereas eight species have a His. Because Cys and His residues are often involved in specific functions within protein structures [34], replacements in this position could be functionally relevant. Four PSSs (2530, 2572, 2574, and 2884) locate within the interaction region with SEM1, a gene involved in DNA damage repair and cell cycle progression [35]. Among these, residues 2530, 2572, and 2574 cluster in the helical subdomain that interacts with FANCD2 [36], a partner of the FA/BRCA complex (also a PSG). Residue 2884 sits in the first Oligosaccharide binding (OB domain. Several PSSs were found accumulated in a disordered segment of the C-terminal region (3363–408) with no documented activity. We observed some PSSs mapped in close proximity to natural variants predisposing to human cancer [28] (Figure 4A). The stop-gained variant Y792*, associated with pancreatic adenocarcinoma (PAAD), is close to three PSSs. The PSS 1158 is in close proximity to the stop-gained variant Q1037*, also associated with PAAD. The selected residue 1646 is flanked by the stop-gained variants S1630* and Y1655*, associated with ovarian cancer and head-neck squamous carcinoma, respectively. The PSS in 1708 maps close to the pathogenic mutation Y1762* associated with ovarian cancer. In the intervening regions between the BRC6-BRC7-BRC8 repeats, we detected a clustering of three PSSs (1913, 2035, and 2037) with two pathogenic variants predisposing to breast cancer (E1953* and S1955*) and the mutation K2013* related to ovarian cancer. Three PSSs (2530, 2572 and 2574) and three pathogenic variants (R2494* associated with bladder urothelial carcinoma, R2520* with ovarian cancer, and W2626 with rectum adenocarcinoma) were found co-localized in the helical domain.

*PTPRC* encodes a tyrosine phosphatase also known as CD45 that regulates T- and B-cell antigen receptor signaling and is associated with oncogenic transformation through changes in expression [37]. We detected 36 and 52 PSSs under M2a and M8, respectively (Table 1). Remarkably, the 52 M8 PSSs concentrate on the extracellular region involved in T cell receptor activation [38], whereas the cytoplasmic segment, which contains the phosphatase domains, seems to be under strong purifying selection (no PSSs; Figure 4B). Within the extracellular region (positions 26–577 of human PTPRC), the PSSs cluster in the cysteine-rich (CR) domain and across the three Fibronectin type 3 (FN3) domains (Figure 4C). We did not find information about germline variants in PTPRC associated with cancer.

## 4. Discussion

### 4.1. Cancer Genes Show Relatively Low dN/dS Values

Because human cancer genes are generally involved in essential cellular functions such as DNA repair, regulation of cell cycle, and apoptosis, strong purifying selection removing deleterious germline mutations is expected, resulting in a dN/dS << 1. On average, the estimated dN/dS ratio across cancer genes (0.12) was somehow lower than previous estimates obtained from mammalian genomes, which yielded values between 0.15 and 0.22 [6,39,40,41]. On the other hand, Thomas et al. obtained a lower dN/dS for cancer-related genes (dN/dS = 0.079) than for other disease-related genes (dN/dS = 0.101) or for non-disease-related genes (dN/dS = 0.100), when comparing humans and rodents [42]. Our results also concord with Blekhman et al., who found significantly lower dN/dS values (dN/dS = 0.061) in human genes associated with cancer compared to genes involved in Mendelian (dN/dS = 0.133) and complex diseases (dN/dS = 0.203) [43].

### 4.2. Positive Selection on Human Cancer Genes is Associated with Hereditary Cancer and Recessive Mutations

Human cancer genes bearing only germline mutations in COSMIC yielded higher dN/dS ratios and higher proportion of PSGs than human cancer genes with only somatic mutations. This result suggests that genes associated with hereditary cancer have less selective constraints than those genes related to sporadic cancer. Indeed, it is expected that slightly deleterious (and advantageous) mutations under weak purifying selection reach higher frequencies in the populations than under strong purifying selection [44]. Therefore, it is expected that variants under relaxed purifying selection are more likely associated with hereditary cancer than with sporadic cancer, as mutations in the latter are expected to be highly deleterious and are therefore removed by purifying selection. In addition, since cancer is a complex and often a late-onset disease, where each allele contributes to a small fraction of cancer risk, it is plausible that variants directly associated with cancer susceptibility are under relaxed selective constraints [43,45,46]. COSMIC does not provide information about the exact number of germline and somatic mutations in each gene category, so we could not evaluate the impact of mutation burden on selective constraints.

We detected that genes associated with recessive mutations show significantly higher dN/dS values and were more often positively selected than genes associated with dominant mutations. It is expected that genes with dominant effects are subjected to stronger purifying selection than those with recessive effects, as deleterious mutations in the former will have immediate disadvantages in heterozygosity. It has been reported that genes with recessive disease mutations have higher dN/dS than genes with dominant disease mutations [43,47]. This result is not independent from the one just discussed, as almost all cancer genes with germline mutations in our dataset were associated with recessive inheritance. Because genes associated with germline mutations and dominant inheritance are unrepresented in our dataset, we were unable to test the potential interaction between mutation type and inheritance mode.

### 4.3. Lack of Variation in Selection across Tissues or Cancer Gene Role

Our analysis did not reveal significant differences in the dN/dS ratios or the proportion of PSGs among tissue types (epithelial, lymphoid, mesenchymal, and others) or cancer role (fusion genes, oncogenes, and TSGs). This might suggest that selective pressures on human cancer genes along the mammal lineage are not directly related to cancer. Since blood and bone marrow cancers are promoted by alterations in the immune system, and genes involved in immunity often undergo fast adaptation [6,7,8], we would have expected a stronger signal of positive selection on genes associated with lymphoid cancers. A previous study on the evolution of cancer genes in hominoids identified a higher dN/dS in TSGs with respect to oncogenes [11]. Although the difference was not statistically significant, we observed a higher average dN/dS for TSGs than for oncogenes. These patterns might be attributed to TSGs being more enriched in genes with recessive mutations (109 out of 206, 52.9%, TSGs carry recessive mutations) than oncogenes (15 out of 208, 7.2%). Nonetheless, the proportion of PSGs was very similar between fusion genes and TSGs (Figure 3C), while the former included very few genes with recessive mutations (8 out of 225, 3.5%). Therefore, although the inheritance factor might influence the strength of purifying selection, positive selection is likely driven by other properties.

### 4.4. Signalling Pathways and Biological Functions of Cancer Genes under Positive Selection

Within the putative list of PSGs, we found an enrichment of genes involved in DNA repair, with a high presence of genes involved in the Fanconi Anemia (FA)/BRCA pathway. Evidence of adaptive evolution in some components of the FA/BRCA pathway (*BRCA2*, *CHEK2*, *FANCC*, *FANCB*, *FANCD2*, and *FANCE*) has been previously identified in mammals [48]. In addition to these genes, we also identified signatures of positive selection in *FANCA* and *FANCG*. The systematic positive selection observed on the FA/BRCA complex might suggest a mechanism of coevolution to maintain the interactions among partners of this network [48,49]. Positive selection on the FA/BRCA complex could be driven by different selective pressures. On one hand, positive selection on this DNA repair pathway could favor a molecular mechanism of tumor resistance to counteract the increased cancer risk associated with longevity [50]. This hypothesis is supported by the signature of positive selection of some FA/BRCA components (*BRCA2*, *FANCA*, *FANCE*, and *FANCL*) identified in long-lived and cancer-resistant species [51,52]. On the other hand, germline variants in the FA/BRCA repair pathway have been associated with hereditary breast–ovarian cancer and Fanconi Anemia in humans [30,32]. Therefore, it is possible that, at least a portion of selected alleles in the FA/BRCA pathway could be pleiotropic and have deleterious late-onset effects like higher cancer risk. Future biochemical characterization of mutants and genetic association studies will help to better understand the consequences of positive selection on the components of the FA/BRCA pathway.

As mentioned in the introduction, antagonistic pleiotropy between positive selection associated with organismal fitness and increased cancer risk could be a general mechanism behind the long-term molecular adaptation of human cancer genes [6,7,8,10]. Immune response, placentation, and spermatogenesis, expected to be shaped by pathogen–host coevolution, maternal–fetal interactions, and sexual selection, respectively, are biological processes also often associated with positive selection on cancer genes [9,10]. Interestingly, we observed a high proportion of immunity-related genes under positive selection, with enrichment in the process of T cell proliferation, which would support the hypothesis of pathogen–host interactions driving adaptive changes of cancer-related genes. At the same time, the identification of several positively selected genes expressed in testis (such as *BRIP1*, *BUB1B*, *KTN1*, and *RANBP2*) is also concordant with the hypothesis that the genetic pathways of spermatogenesis, which evolve in response to sexual selection and intrasexual conflict, often coincide with those used by cancer cells to increase their survival and replication [6,9,10].

### 4.5. Functional Relevance of Residues under Positive Selection in Cancer Genes

We identified 25 PSSs in BRCA2, where some positions mapped close to natural variants associated with cancer. A similar signature of positive selection in BRCA2 was previously identified by O’Connell [48]. Although no PSS matched with pathogenic variants, which is not surprising since cancer is not a driver of species adaptation, they might be involved in cancer susceptibility or tumor resistance, especially when located in regions of functional importance. Future biochemical studies will help to assess the evolutionary significance of variants in PSSs. It is worth to mention that, although we focused on the potential effect of mutations in PSSs, it is expected that most of the mutations associated with cancer risk fall on sites under strong purifying selection during species evolution. Therefore, in the future, it would be interesting to consider the most conserved domains of human cancer genes as potential candidate disease regions.

## Figures and Tables

**Table 1:** List of cancer genes showing evidence of positive selection.

Gene	Function	dN/dS
*IL2*	T cell proliferation and regulation of the immune response.	0.748
*FAS*	Apoptosis	0.601
*FCRL4*	B cell receptor signaling	0.601
*NUTM2A*	Unknown	0.574
*PALB2*	Tumor necrosis factor, apoptosis	0.507
*PDCD1LG2*	T cell proliferation; immune response	0.506
*FANCG*	Fanconi Anemia (FA) group; DNA repair	0.500
*BRCA2*	Double-strand break repair and/or homologous recombination	0.468
*CD274*	T cell effector regulation; attenuation of anti-tumor immunity	0.465
*FANCC*	F.A. group; DNA repair	0.445
*CASP8*	Protease inhibitor; apoptosis	0.363
*PTPRC*	Protein phosphatase; receptor; immune response	0.361
*FANCD2*	F.A. group; DNA repair	0.348
*BARD1*	Control of the cell cycle in response to DNA damage	0.333
*ERCC5*	DNA repair	0.321
*NCOA4*	Androgen receptor signaling	0.312
*NIN*	Centrosome localization	0.307
*BRIP1*	Double-strand break repair and/or homologous recombination	0.286
*COL1A1*	Collagen component	0.276
*CD79B*	B cell differentiation and activation	0.275
*BLM*	Basic helix-loop transcription factor	0.264
*CD79A*	B cell differentiation and activation	0.253
*PMS2*	DNA binding protein	0.225
*KTN1*	Kinesin-driven vesicle motility; cadherin binding	0.211
*PRF1*	Apoptosis; immune response	0.197
*SET*	Chaperone; phosphatase inhibitor	0.180
*ARHGEF12*	Regulation of RhoA GTPase	0.178
*CHEK2*	Checkpoint-mediated cell cycle arrest, activation of DNA repair and apoptosis	0.175
*PTPRB*	Protein phosphatase; receptor; angiogenesis	0.156
*SS18*	Chromatin-binding protein; transcription regulation	0.153
*FLT3*	Regulation of apoptotic process	0.144
*COL2A1*	Collagen component	0.132
*MLLT6*	Nucleic acid binding; zinc finger transcription factor	0.130
*KDM6A*	Transcription factor; chromatin remodeling	0.120
*POU2AF1*	Transcriptional coactivator; immune response	0.106
*MED12*	Nucleic acid binding; transcription cofactor	0.097
*RBM15*	RNA binding protein	0.088
*RABEP1*	Membrane fusion; apoptosis	0.086
*BRAF*	Transduction of mitogenic signals; apoptosis	0.079
*PICALM*	Vesicle coat protein	0.068

## Data Availability

The data and code required to reproduce the results of this study are available in https://github.com/avicens/cancer_genes_selection.

## Supplementary Materials

The following are available online at http://www.mdpi.com/2073-4425/9/12/582/s1, Figure S1: Mammal phylogenetic tree of mammalian species assembled for this study, Figure S2: Global dN and dS values according to mutation type (A,B) and inheritance (C,D), Figure S3: (A) Correlation between protein sequence length and global dN/dS estimates; (B) Comparison of protein length between positively selected and not selected genes; (C) Comparison of protein length among mutation-type categories. Statistical *p*-values are shown for multiple and pairwise comparisons. Mean of sequence length is indicated below each category in (B,C), Figure S4: Proportion of positively selected sites across COSMIC categories: (A) mutation type; (B) genetic dominance; (C) tissue type; and (D) cancer role. The number of genes in each category is indicated within each square. Significance levels for chi-square tests are indicated below each plot: non-significant (ns) and significant *p*-value < 0.05 (*). Abbreviated categories: Som + Ger: Genes bearing both somatic and germline mutations; TSG: tumor suppressor genes. Table S1; List of Cancer Gene Census of COSMIC genes used in the study. Table S2: Genes discarded from the analysis in the different pre-processing steps, Table S3: Genes discarded from the analysis in the different pre-processing steps, Table S4: Protein Ensembl accessions of all orthologues retrieved in this study, Table S5: Evolutionary parameters estimated for each gene.
