# Supplementary material for: Selective Pressures on Human Cancer Genes along the Evolution of Mammals
Source: Genes (Basel). 2018 Nov 28;9(12):582. doi: 10.3390/genes9120582 (PMC6316132; doi:10.3390/genes9120582)
Supplement: Supplementary file 1 [file genes-09-00582-s001.zip › genes-389232-supplementary-figures-final.docx]

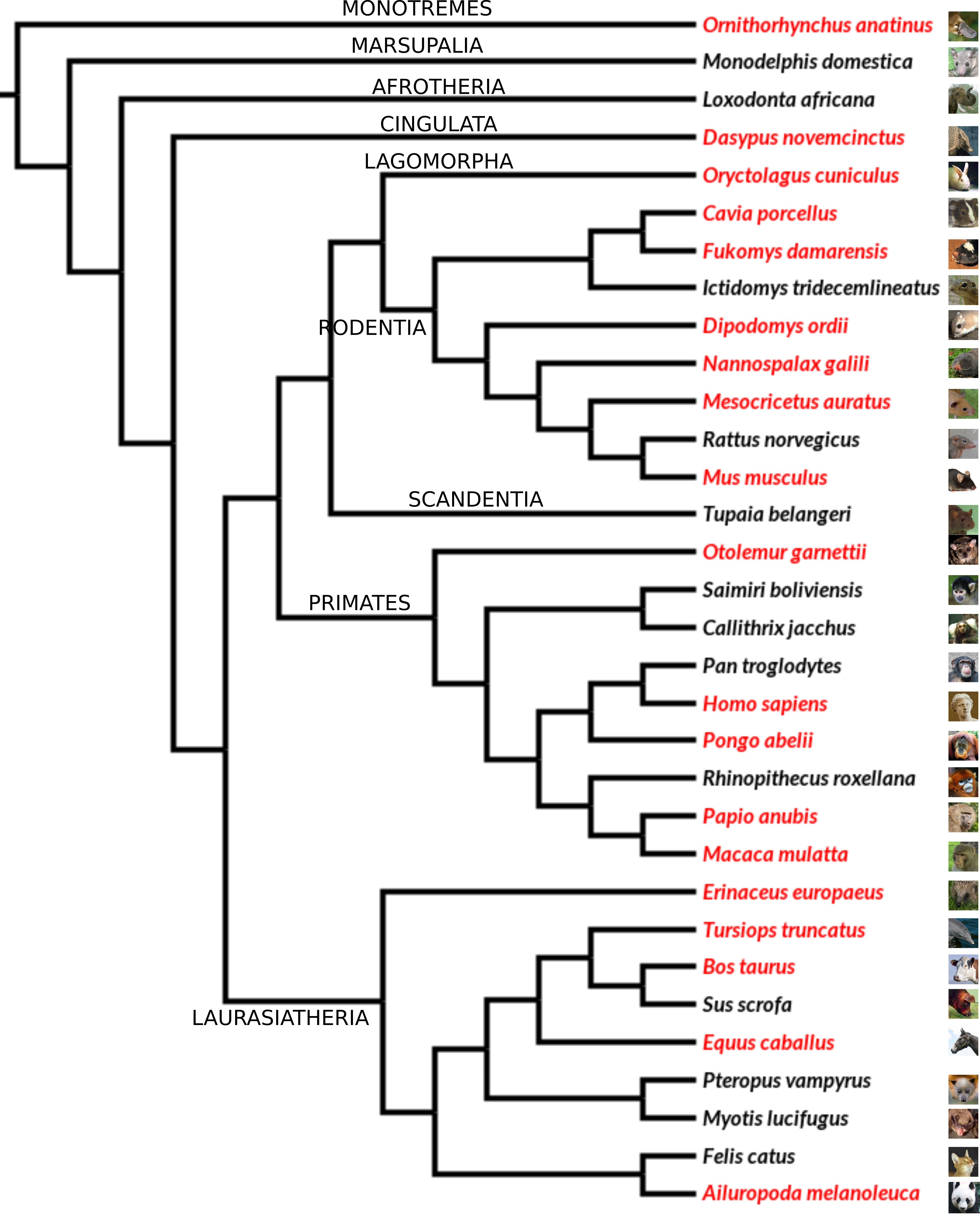


**Figure S1.** Mammal phylogenetic tree of mammalian species assembled for this study.


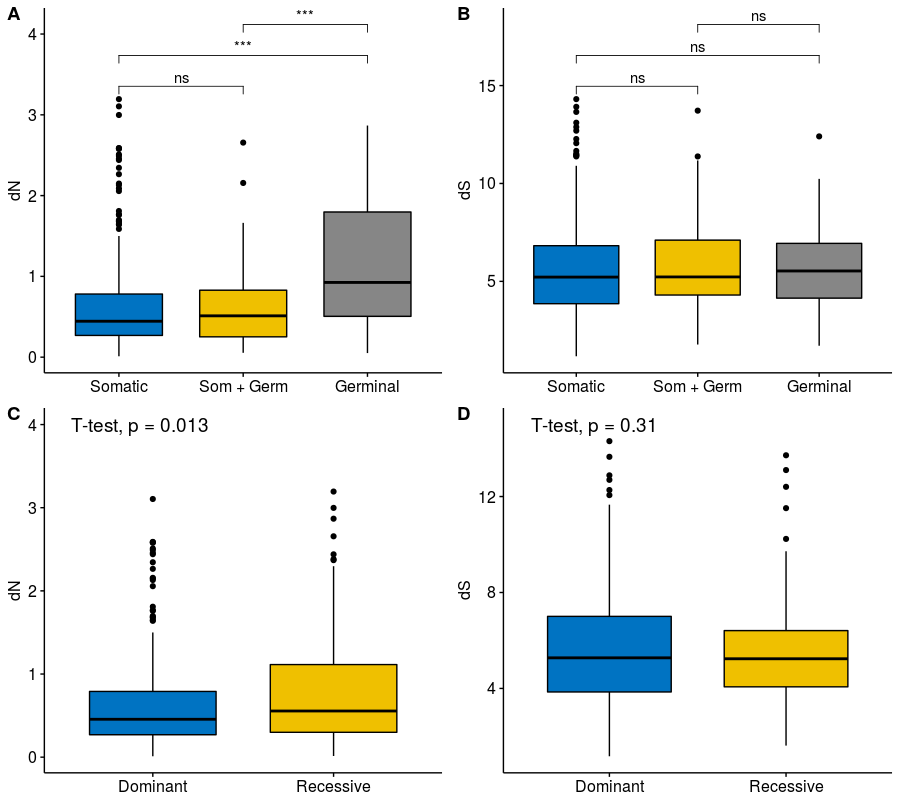


**Figure S2.** Global dN and dS values according to mutation type (A,B) and inheritance (C,D).


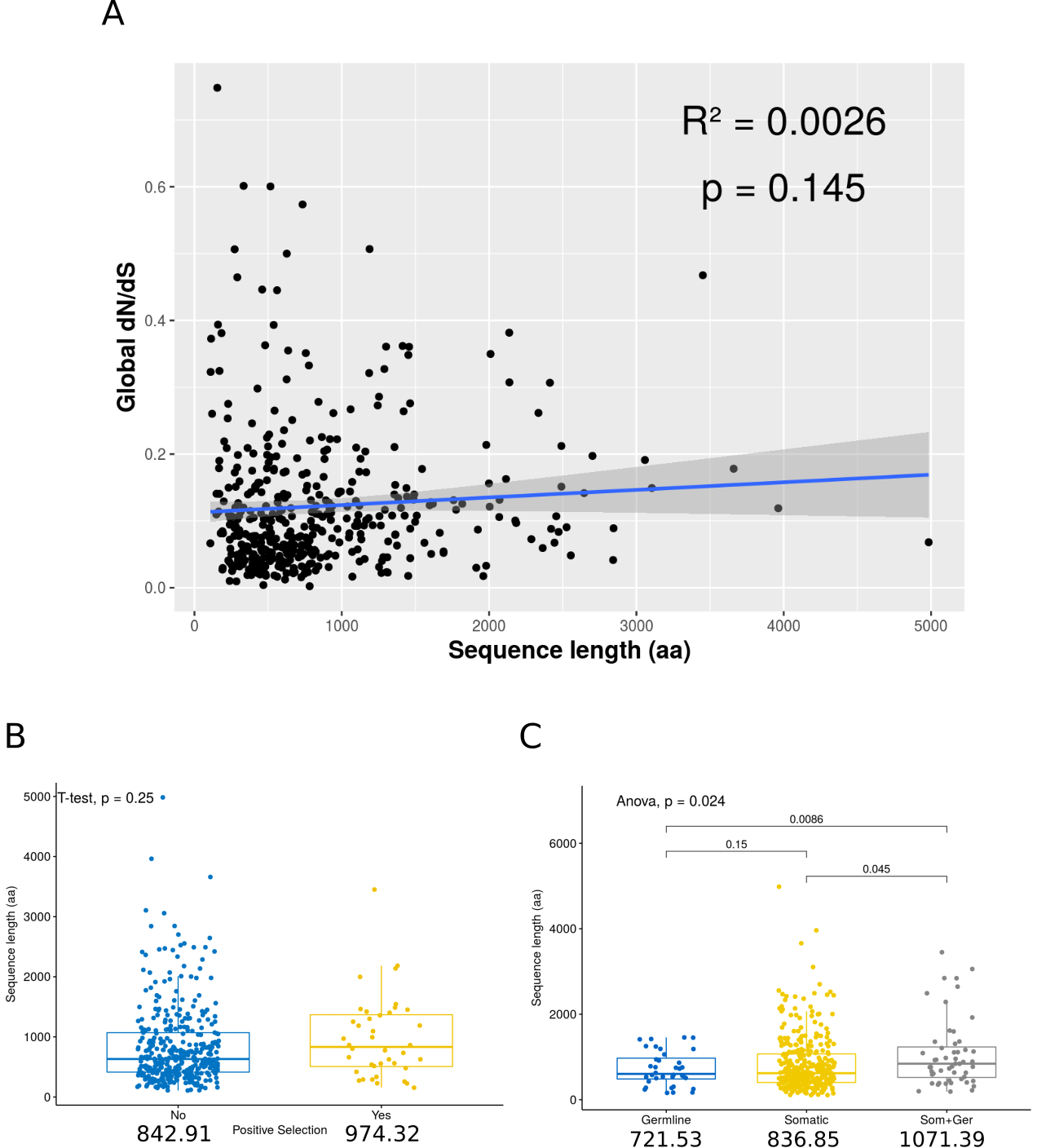


**Figure S3.** (A) Correlation between protein sequence length and global dN/dS estimates; (B) Comparison of protein length between positively selected and not selected genes; (C) Comparison of protein length among mutation-type categories. Statistical *p*-values are shown for multiple and pairwise comparisons. Mean of sequence length is indicated below each category in (B,C).


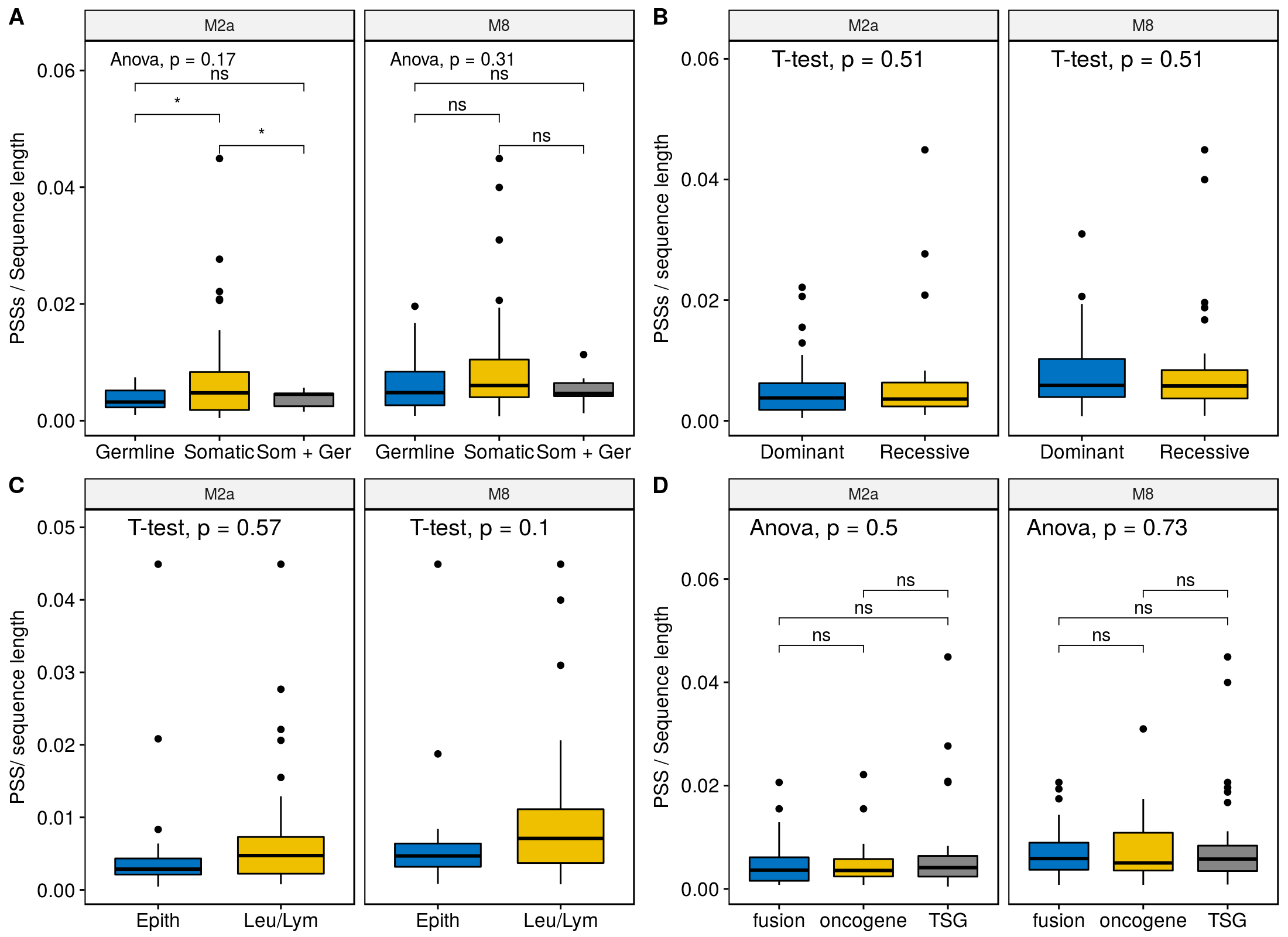


**Figure S4.** Proportion of positively selected sites across COSMIC categories: (A) mutation type; (B) genetic dominance; (C) tissue type; and (D) cancer role. The number of genes in each category is indicated within each square. Significance levels for chi-square tests are indicated below each plot: non-significant (ns) and significant *p*-value < 0.05 (*). Abbreviated categories: Som + Ger: Genes bearing both somatic and germline mutations; TSG: tumor suppressor genes.
