# Supplementary material for: Selective Pressures on Human Cancer Genes along the Evolution of Mammals
Source: Genes (Basel). 2018 Nov 28;9(12):582. doi: 10.3390/genes9120582 (PMC6316132; doi:10.3390/genes9120582)
Supplement: Supplementary file 3 [file genes-09-00582-s001.zip › TableS2_discarded_genes.odt]

Table S2. Discarded genes performing the evolutionary analysis.

| Criteria | Genes |
| --- | --- |
| Human transcripts have a TSL < 1 | ACKR3, AFF3, CEBPA, FANCF, FAT1, FOXL2, HIST1H3B, HIST1H4I, HLA-A, HOXD11, HOXD13, IRS4, JAK1, JUN, KMT2D, MTCP1, NUTM2B, PIK3CA, POT1, SMAD4, SOX2, TAL2 |
| MSA with < 10 ortholog sequences after filtering | AFF1, AMER1, ARID1B, ASXL1 , ATP1A1, BRCA1, CACNA1D, CIC, CNTRL, CUX1, EZR, FBXO11, FUBP1, GAS7, GNAS, KMT2C, LRP1B, MAP2K4, MAP3k13, MITF, MKL1, MSI2, MTOR, MUC1, MUTYH, NRG1, NTRK3, NUP214, NUTM1, OLIG2, PLCG1, POLQ, PPARG,  PTCH1, PTPRK, QKI, RAP1GDS1, RARA, RNF213, RUNX1T1, SFPQ, SMARCA4, SSX1,SSX2,SSX4, TAL1, TCF7L2, TFEB, TNFRSF14, TRRAP, TSC2, UBR5, ZFHX3 |
| Gene trees with nRF distance < 0.6 compared with the species tree | BAX, BCL11A, BTG1, CBFB, CD74, CHCHD7, DNM2, ETV1, FBXW7, FCGR2B, FGFR2, H3F3B, KRAS, LEF1, LMO1, LMO2, MAX, MLF1, PAX7, PRRX1, RAC1, RSPO3, SMARCE1, SOCS1, TBL1XR1, TPM4, ZRSR2 |

TSL: Transcript Support Level

MSA: Multiple Sequence Alignment

nRF: normalized Robinson-Foulds distance
